# Editorial: Advances in social cognition assessment and intervention in autism spectrum disorder

**DOI:** 10.3389/fpsyt.2022.962843

**Published:** 2022-08-26

**Authors:** Soumeyya Halayem, Asma Bouden, Isabelle Reine Amado, Bennett Leventhal

**Affiliations:** ^1^Faculty of Medicine, Tunis El Manar University, Tunis, Tunisia; ^2^Department of Child and Adolescent Psychiatry, Razi Hospital, Manouba, Tunisia; ^3^Centre Hospitalier Sainte-Anne, Paris, France; ^4^School of Medicine, University of California, San Francisco, San Francisco, CA, United States

**Keywords:** autism, social cognition, cultural aspects, assessment, intervention

Social cognition (SC) refers to all cognitive processes and skills involved in social interactions, that needs to be engaged in a perpetual changing environment. SC encompasses several domains: theory of mind (ToM), facial emotions processing, empathy, emotional regulation, and attributional style etc. Numerous are altered in patients suffering from autism spectrum disorders (ASD) resulting, in addition, or in combination with ASD core symptoms, as it will be discussed in this Research Topic, significant social impairments. These latter needs better assessment, new theoretical approaches as well as innovative interventions. Since 30 years, tools of assessment were created and adapted to cultural characteristics. Progressively, SC has been a crucial therapeutic target for this population leading to the development of therapies using technological advances. Interventions are now available with encouraging results that require confirmation. The goal of this Research Topic was to improve our understanding of SC deficits in people suffering from ASD and search for new methods of assessment. Relevant empirical and theoretical articles were selected. Subtopics include assessments considering assessment, contribution of new technologies in therapeutic interventions, and etiopathology of SC in ASD.

## Assessment

### New SC assessment tools and correlations with cognitive functioning

Three articles present new tools for assessment of SC. Two of them deal with the validation of a new facial emotion recognition tool for children, developed by a Tunisian team whose originality consists in the use of photos of three levels of difficulty and videos of the basic emotions of Ekman expressed by actors of different gender and age. The first reliability study (Taamallah et al.) showed good internal consistency for each subtest and normative references for typically developing (TD) children of 7–12 years. The second one (Jelili et al.) confirmed the clinical validation of the tool in children with high functioning autism.

Morel-Kohlmeyer et al. presented a new battery for adults SC evaluation named “ClaCoS,” which allows testing: emotion recognition, ToM, attributional style, and social perception and knowledge; but also subjective complaints in SC. In this study, the ASD group showed deficits in all domains of SC and reported greater complaints regarding their social abilities, that were correlated to neuropsychological performances. Along the same lines, Peñuelas-Calvo et al. focused on the links between intelligence quotient using the WISC and Reading The Mind in the Eyes Test performances' in children and adolescents with Asperger's syndrome. Intelligence quotient and age were positively correlated to the performance in ToM.

Briot et al., systematically reviewed new technologies for the assessment of emotional facial expression in people with ASD through 20 studies. Authors found discrepancy between the expressed emotions and the expected ones with low coordination of facial muscles. The hypothesis of a poor correspondence between mental representations and facial emotion expressions underlined by an ≪ alteration of sensory-motor neural circuit involved in the processing of information ≫ was raised.

### Eye attention to social stimuli

Four studies looked at the assessment of social attention through eye-tracking procedures. Bochet et al. explored the processing of emotional and neutral faces using an eye-tracking paradigm with pre-school aged children with ASD and age-matched TD children. Participants with ASD make fewer fixations to emotional faces than their TD peers, and equivalent duration of first fixation on emotional faces to that on neutral faces. Participants also demonstrated neither habituation nor increased interest in the changing emotional expressions over the course of the task. These results confirm literature reports of a lesser interest to emotional faces and a lack of sensitivity to changes in facial expression in children with ASD.

Mouga et al. compared visual attention to faces vs. objects, in ecological situations using three tasks from the ADOS. Participants with ASD's focused less and shorter on faces and their performances became similar to TD children when they were asked to tell a story from a photo. This study has a therapeutic interest in remedying deficits in spontaneous social attention.

Wang et al. used a preferential looking paradigm using eye gaze tracking devices when presenting to children with ASD at the same time two stimuli: a photo of eyes and a photo of objects alternating with a high attention attracting object in participants diagnosed with ASD (vehicle) vs. a low one (vegetable). The results showed that preference and interest in the eyes decreased among ASD patients' when the competitive stimulus was a high attracting object. This study has interesting therapeutic implications for improving eye contact in this population by using objects as stimuli to motivate joint attention.

The last two studies by Ioannou et al. compared social visual attention in four groups: patients with ASD, ASD and ADHD, ADHD, and a neurotypical group using a static medium with real social situations of low and high complexity. Authors found a significant lower duration of face fixation with a lengthening of the time of first fixation in the ASD-ADHD vs. the ASD group. The duration of face fixation was reduced in ASD vs. TD, especially for scenes with high social complexity. Regarding nonsocial clues, Iaonnou et al. found that there was no difference between the groups.

These two studies raise the hypothesis of possible biomarkers that can distinguish the different conditions (ASD, ADHD, ASD with ADHD and TD) with an interest for early diagnosis and specific therapeutic interventions.

## Interventions in SC

The first article made a link between assessment and therapeutic effects on SC in ASD. In their perspective article, Yamamoto et al. examined the usefulness of visualizing face-to-face interaction in social skills learning scenes for children with ASD using a business microscope that can qualify and visualize face-to-face interactions automatically. This device's use may provide an objective measurement that complements the observer's evaluation in intervention's validation programs.

Dandil et al. conducted a synthesis of all studies of cognitive remediation interventions for adolescents and adults with ASD using the PRISMA statement. They concluded, on 13 studies, that cognitive remediation therapies and interventions could potentially be effective in improving SC and functioning. However, methodological challenges were a source of heterogeneity. This calls for double-blind randomized studies of longer duration, assessing the effect size and generalization of improvement in SC as well as social functioning and the possible linked effects between these latter.

Herbrecht et al. studied the two-years outcomes of a short-term, highly, intensive early 18-day family intervention in a case series study without control group. The therapy consists of up to 6 h of play sessions per day involving the family. Parents are intensively coached while playing with their child and while observing their child's behavior *via* video observation of play sessions. Authors found, for 32 children with a mean age of 44 months, presenting moderate to severe ASD, that ADOS total scores decreased significantly from baseline to 2-year follow-up. The DD-C-GAS, fullfilled by parents, showed significant improvements on all subdomains in areas of everyday functioning. There was a significant relationship between parents' treatment adherence and ADOS scores. These results invite to strengthen early intensive approaches within randomized studies.

In a perspective article, Bochet et al. brought some light on their clinical experience to adapt the PEERS© model for adolescents into French after reviewing its limitations and its positive aspects in generalizing SC competencies, when involving parents. Indeed, follow-up measures over up to 5 years have shown improved SC. This maintenance could be explained by the participation of parents as “social coaches” and needs randomized double blind studies for confirmation.

Dubreucq and Dubreucq conducted a systematic research following PRISMA guidelines, on autistic women's needs for care relating to romantic relationships and reproductive health. They found eight studies on interventions addressing the first domain and no study on reproductive health or parenting. Most interventions did not deal with gender-sensitive content though women with ASD face challenges in theses domains.

Fu et al. studied the predictors of change in a 12 weeks play-based communication and behavior intervention for 70 13–30-month-old toddlers at high-risk for ASD. Hierarchical regression analysis concluded that parenting stress, level of completion of training at home and mother-child dyadic synchrony were crucial factors in predicting the intervention's efficacy. These factors are working levers to improve the prognosis of early interventions involving parents.

In their systemic review, including 39 studies (N = 1,774 participants), Mayer-Benarous et al. explored music therapy for children with ASD and/or other neurodevelopmental disorders. Positive effects of educational music therapy on patients with ASD was reported in most controlled studies on speech production and of improvisational music therapy on social functioning. Authors concluded that improvisational music therapy appears relevant for individuals with both ASD and intellectual disability.

## Etiopathology

### Networks

Sato et al., using fMRI, showed that modulation from the amygdala to the neocortex during observation of dynamic facial expressions is reduced in an ASD group. Based on these findings and literature review, they hypothesized that weak modulation from the amygdala to the neocortex—through which emotion modulates various types of perceptual, cognitive, and motor processing functions—underlies the social atypicalities in people with ASD.

Pino et al. aimed to map the network of SC domains in 76 children with ASD matched for verbal age to TD children through graph analysis using three measures: Eyes Task-Simplified, Comic Strip Task and Social Information Processing Interview (SIPI). TD children exhibited a SC network characterized by a single domain, with nodes of higher closeness and strength compared to the ASD network. For instance, the node related to encoding in the SIPI was not connected in the ASD network. This suggests that people with ASD can encode social information, but that this latter would not be utilized by the entire network.

### Looking for SC related biomarkers of ASD

Based on the finding that people with ASD show an atypical pattern of autonomic nervous system activation (ANS) induced by psychosocial stimulation, Doi et al. proposed a new assessment tool to evaluate the risk of ASD by measuring ANS activation in response to emotional stimulation. Authors video-recorded faces of 17 and 21 adult males with and without ASD while watching emotion-inducing video clips. Though the performance was modest, the gradient succeeded in classifying people with and without ASD. Taking into consideration the fact that the study recruited only high-functioning adults who may use compensatory strategies, ASD screening by measurement of pulse wave could be promising to evaluate ASD risk.

Bearing in mind that it is generally challenging to assess ASD individuals using task-based and fMRI paradigms, Yao et al. reviewed neuroimaging studies examining inter-hemispheric resting state functional connectivity (rsFC) abnormities. There are converging findings on reduced inter-hemispheric rsFC in brain networks particularly in posterior hubs and in the corpus callosum. Thus, the strength of inter-hemispheric rsFC may be a promising biomarker for assisting in ASD diagnosis.

All these results remain in the field of research, as biomarkers in functional imaging and ANS assessment are within the reach of few clinicians.

### Unified theories

Crespi developed a theory unifying ASD semiology and etiology through pattern concept. Pattern is defined as ≪ a repeated configuration, with recurring, ordered, or predictable characteristics, and discernable interrelationships of components ≫. This concept is related to information processing, including ≪ sensory input, entropy, and predictability ≫. ASD involves enhanced ≪ pattern perception, recognition, maintenance, generation, processing, and seeking ≫. Restricted interests and repetitive behavior result from increased and imbalanced pattern-related perception and cognition, and social deficits result in part from the lack of clear pattern in social interactions, with non-pattern avoidance of social interaction, combined with the interference of restricted interests with social development. This approach has strong implications for research as well as personalized therapy.

## Conclusion

This Research Topic helps highlighting new tools of SC assessment, with spot on facial emotion recognition, and deficits of interest to facial stimuli in ASD population. FMRI, ANS and eye tracking specificities were proposed as biomarkers calling to confirmatory works studying correlations with clinical phenotypes. The link between symptoms of ASD and SC deficits in the light of pattern conception may be an interesting framework to develop research approaches.

## Author contributions

All authors listed have made a substantial, direct, and intellectual contribution to the work and approved it for publication.

## Conflict of interest

The authors declare that the research was conducted in the absence of any commercial or financial relationships that could be construed as a potential conflict of interest.

## Publisher's note

All claims expressed in this article are solely those of the authors and do not necessarily represent those of their affiliated organizations, or those of the publisher, the editors and the reviewers. Any product that may be evaluated in this article, or claim that may be made by its manufacturer, is not guaranteed or endorsed by the publisher.

